# web cellHTS2: A web-application for the analysis of high-throughput screening data

**DOI:** 10.1186/1471-2105-11-185

**Published:** 2010-04-12

**Authors:** Oliver Pelz, Moritz Gilsdorf, Michael Boutros

**Affiliations:** 1 German Cancer Research Center (DKFZ), Div Signaling and Functional Genomics and University of Heidelberg, Faculty of Medicine Mannheim, Dept Cell and Molecular Biology, Im Neuenheimer Feld 580, D-69120 Heidelberg, Germany

## Abstract

**Background:**

The analysis of high-throughput screening data sets is an expanding field in bioinformatics. High-throughput screens by RNAi generate large primary data sets which need to be analyzed and annotated to identify relevant phenotypic hits. Large-scale RNAi screens are frequently used to identify novel factors that influence a broad range of cellular processes, including signaling pathway activity, cell proliferation, and host cell infection. Here, we present a web-based application utility for the end-to-end analysis of large cell-based screening experiments by cellHTS2.

**Results:**

The software guides the user through the configuration steps that are required for the analysis of single or multi-channel experiments. The web-application provides options for various standardization and normalization methods, annotation of data sets and a comprehensive HTML report of the screening data analysis, including a ranked hit list. Sessions can be saved and restored for later re-analysis. The web frontend for the cellHTS2 R/Bioconductor package interacts with it through an R-server implementation that enables highly parallel analysis of screening data sets. web cellHTS2 further provides a file import and configuration module for common file formats.

**Conclusions:**

The implemented web-application facilitates the analysis of high-throughput data sets and provides a user-friendly interface. web cellHTS2 is accessible online at http://web-cellHTS2.dkfz.de. A standalone version as a virtual appliance and source code for platforms supporting Java 1.5.0 can be downloaded from the web cellHTS2 page. web cellHTS2 is freely distributed under GPL.

## Background

High-throughput cell-based screens have become an important experimental tool for the analysis of many cellular processes. Whole genome sequences and methods for gene silencing by RNA interference (RNAi) have enabled loss-of-function analysis in *ex vivo *and *in vivo*, opening new avenues for functional analysis that were previously unfeasible [1,2]. Different experimental methods to assess phenotypic changes are being used, from single-channel homogenous readouts to multi-channel cytometry and imaging, producing large data sets that need to be analyzed to extract phenotypically relevant information. RNAi screening has found a broad user-base as a genetic method to dissect many different cellular processes, such as cell survival, signaling pathways and other cellular phenotypes in a high-throughput manner [3-6].

High-throughput screens are mostly performed using 96- to 384-well plates and produce large data sets that need to be normalized, summarized and ranked to generate a list of significant phenotypic modifiers. Large-scale RNAi screens can easily exceed more than 100,000 data points per screening experiment and specialized statistical approaches have been developed for their analysis [7-10]. Quality control assessments of assays and screening data are performed to provide benchmarks for the overall performance, such as experiment-wide performance of controls, reproducibility between replicate experiments, as well as other statistical quality control measures [10-13].

We have previously described cellHTS as an analysis toolbox for cell-based high-throughput screens [7]. cellHTS is implemented in R/Bioconductor [12] as a command-line utility that provides a workflow for the analysis of high-throughput data sets. cellHTS and cellHTS2 have become widely used in the community as they provide an end-to-end solution for the analysis of high-throughput screening data sets, while retaining the flexibility to incorporate further functions for statistical analysis as the field matures. However, an obstacle for general use in the laboratory was the lack of an integrated and easy-to-use solution for the configuration of screening plates, choice of controls and analysis methods.

**Figure 1:**
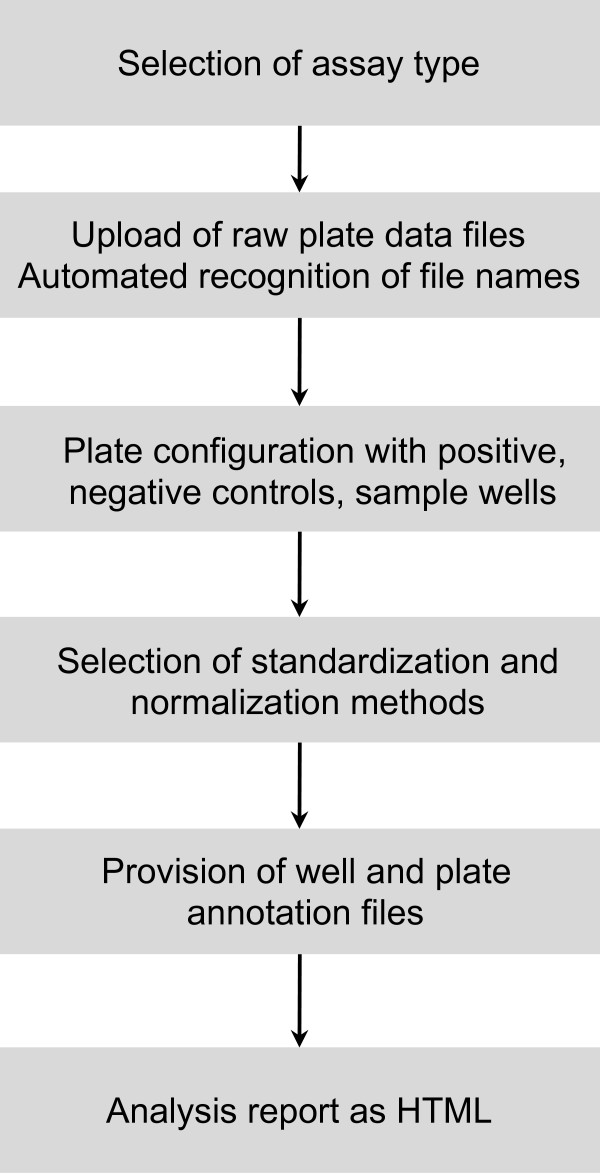
**Schematic representation of the workflow for the analysis of high-throughput screening data sets**.

Here, we present a web-based application that guides the user through all steps required for the analysis of high-throughput screens, including configuration of plates and controls, normalization and statistical quality control (Figure 1). The software allows the user to upload a variety of file formats, select different methods for summarization and normalization, and returns a complete analysis report by E-mail or as a download. Analysis workflows can be saved as templates for re-analysis, or possible submission as supplementary material for publications. The software can be accessed at http://web-cellHTS2.dkfz.de or downloaded as a fully functional Java application (for platforms supporting Java 1.5.0) or VirtualBox appliance.

## Implementation

Data files needed for the analysis are generated through the graphical user interface or can be provided through upload forms. web cellHTS2 also provides an import module that supports upload of a spectrum of different file formats. web cellHTS2 implements error detection mechanisms for each data file or website input, checking for common input errors prior to running cellHTS2. Once the configuration of a screening experiment is completed, the analysis project, containing information on the complete session including all input files and processing parameters, can be saved for re-use. This function allows for rapid re-processing of similar datasets and generation of a full documentation of the analysis. The results of the analysis can be streamed to the web browser or can be sent via E-mail directly.

web cellHTS2 was implemented based on a Java Server Pages infrastructure using the Tapestry5 [14] open-source web-framework, which facilitates maintenance and extension of the web application. The frontend has been designed to run remotely on a Tomcat5 webserver [15] but can also be installed locally using an integrated Jetty [16] Java web-server. AJAX (Asynchronous JavaScript and XML) technology is used to improve the interactivity of web cellHTS2.

**Figure 2:**
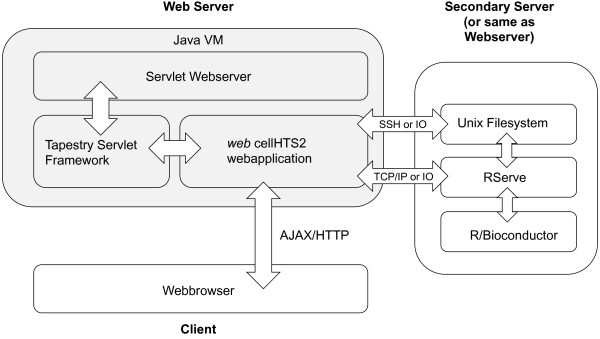
**Implementation of *web *cellHTS2**. The user interacts with the application that resides on an internal or external server using a web-browser interface. The software architecture includes Tapestry as extensible framework and interacts with R using a scalable R-server implementation. The software is available as open-source and can be downloaded from http://web-cellhts2.dkfz.de.

Interaction between the cellHTS2 R/Bioconductor software and the Java based web application is achieved using R-serve, which can be run on an independent webserver. This separates the web application logic from the statistical calculations, thereby reducing the computational load for accessing the webserver and allowing the analysis of several high-throughput screening data sets in parallel (Figure 2). The workload for computational calculations can be scaled at runtime by setting the maximum number of parallel analyses.

## Results

web cellHTS2 facilitates the analysis of high-throughput screening data by providing an easy to use web-application. It has been developed with a view towards large-scale RNAi screens but can also be employed for the analysis of small molecule screens. A particular focus has been to provide a user-friendly interface to select analysis parameters and to generate "re-usable" analysis workflows. Furthermore, error-checking procedures of raw data and annotation files, and automated pre-processing of uploaded data have been implemented. web cellHTS2 can be accessed online or downloaded for local installation. web cellHTS can also be downloaded as a virtual appliance to run web cellHTS2 in a contained environment [17].

**Figure 3:**
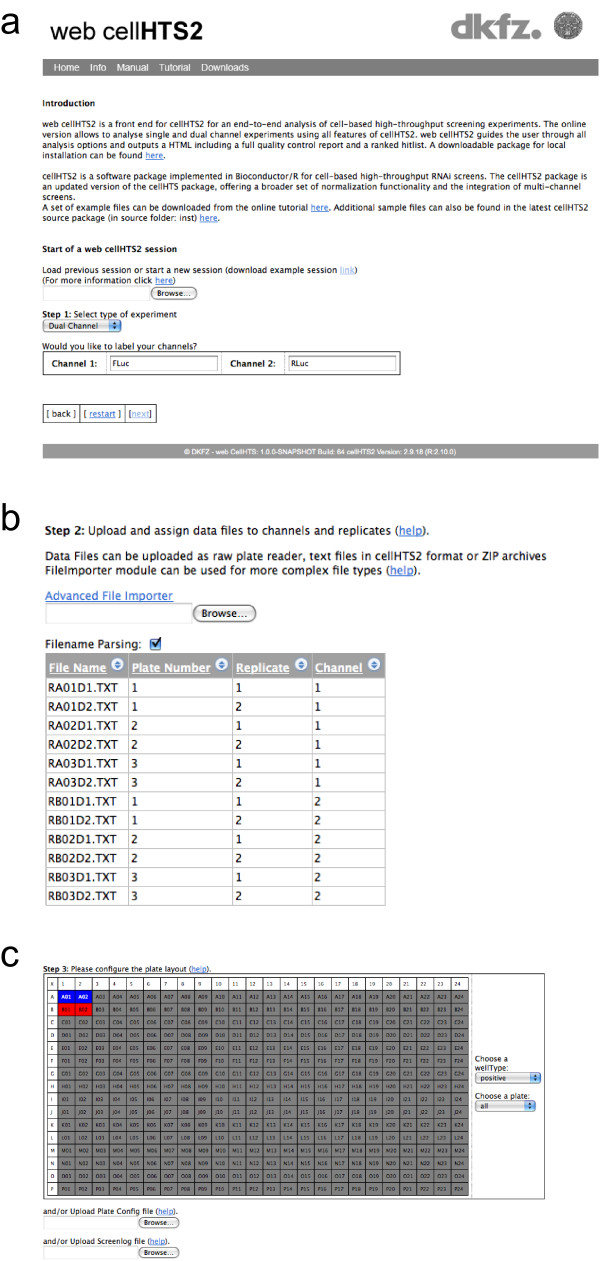
**Screenshots of the analysis workflow of high-throughput screens by web cellHTS2**. (a) The user can start a new analysis or upload previous analysis templates. (b) The data file upload form with parameter editor. (c) Graphical plate configuration editor.

The web application implements three steps in the analysis workflow. In the first step, the user starts a new analysis by choosing the type of experiment (current options are single or dual-channel experiments) or can upload a previously saved workflow (Figure 3a). Uploaded workflows can be modified, e.g. by altering analysis parameters or replacing data files. In the next step, raw data files from high-throughput screening experiments are uploaded. The web application recognizes plate, replicate and channel files, or file assignments can be manually annotated. This feature allows rapid upload of large data sets that can easily entail several hundred data files (such as generated by multi-mode platereaders). Also, previously generated "plate list" files can be uploaded (Figure 3b). An import module can also be used to upload more complex data files. Step three involves configuration of the layout of the multi-well plates which were used in the screening experiment. The graphical user interface allows the user to indicate which wells contain negative, positive and other controls (Figure 3c). Alternatively, previously generated configuration files with existing plate layouts can be uploaded. At this stage the user can select among multiple options regarding how the data is processed and different channels summarized. Table 1 shows a list of options that are currently implemented in cellHTS2, which include both sample and control-based normalization methods, as well as procedures such as B-score normalization [18] to remove spatial artefacts. In the last step, annotation and description files are uploaded or manually described using an edit form. The workflow can then be saved for future analysis or reference. The analysis report is either streamed as a compressed file via HTML or sent by E-mail. A tutorial on the analysis of high-throughput screens with web cellHTS2, including a sample data set, is provided on the website.

**Table 1:** Examples of normalization options

Normalization option	Description
Median	Measurements are divided by the median of all sample wells in the plate

Shorth	The midpoint of the 'shorth' of the distribution of all sample wells is used for normalization

Mean	Measurements are divided by the mean of all sample wells in the plate

Negatives	Measurements are divided by the median of negative controls in the plate

Percent control	Measurements are divided by the mean of the plate's positive control

Normalized percent control	Measurements are divides by the difference of the plates positive and negative controls

B-score	A two-way (row and column) median polish is applied to each plate

Robust local fit regression	Spatial effects are normalized by fitting a bivariate local regression

Loess regression	Spatial effects are normalized using Loess regression

## Discussion

The application presented here is a web or stand-alone program to facilitate the analysis of high-throughput screening data. High-throughput screening experiments are of increasing importance, both for basic science and drug discovery. Such data sets easily exceed the complexity of transcriptome experiments, however there are still comparably much fewer tools available that enable an easy-to-use analysis. cellHTS and other software packages [9] have started to address this issue by enabling an end-to-end analysis of high-throughput screening data sets and have become widely used in the community. Here, we provide a web application as a front-end for cellHTS2 to increase its accessibility and accelerate the analysis of high-throughput screening data sets. The web application can be used both for RNAi and compound screening experiments and can be extended to meet future needs. In contrast to commercial packages, we provide an open-source and extensible solution for online and offline usage.

## Conclusions and future directions

web cellHTS2 provides an intuitive interface for the analysis of high-throughput screens. The user can choose among different options for the analysis of screening data sets. Statistical analysis options will be expanded as new methods become available and broadly used [9,18]. The graphical user interface for the configuration of screening experiments and the option to save "re-usable" session templates make it convenient to use in the laboratory. Future developments of the application will be to provide direct links to phenotype databases [19], e.g. to compare hit lists, to annotate hit list with additional information from public databases e.g. through BioMart and to extend the analysis by functional annotation data such GO enrichment analysis. It is also planned to provide diagnostic plots "on-the-fly" to allow the user to compare different normalization strategies.

## Availability and requirements

Project name: web cellHTS2

Project home page: http://web-cellHTS2.dkfz.de

Operating system(s): Platform independent Programming language: e.g. Java Other requirements: Java 1.5.0

Downloadable Version: R 2.10.0, cellHTS2 2.11.1 and Rserve 0.6.0

Virtual appliance: Open source software *Virtual box *http://www.virtualbox.org

License: GNU GPL Any restrictions to use by non-academics: none

## Competing interests

The authors declare that they have no competing interests.

## Authors' contributions

OP developed the software. MG provided advice in the design and development of the software package. MB conceived the concept and methodology and supervised the project. MB and OP wrote the manuscript. All authors read and approved the final manuscript.

## References

[B1] Boutros M Ahringer J The art and design of genetic screens: RNA interference Nature Reviews Genetics 2008 9 554 566 10.1038/nrg2364 18521077

[B2] Moffat J Sabatini DM Building mammalian signalling pathways with RNAi screens Nature Reviews Molecular Cell Biology 2006 7 177 187 10.1038/nrm1860 16496020

[B3] Flockhart I Booker M Kiger A Boutros M Armknecht S Ramadan N Richardson K Xu A Perrimon N Mathey-Prevot B FlyRNAi: the Drosophila RNAi screening center database Nucleic Acids Research 2006 34 D489 494 10.1093/nar/gkj114 16381918 PMC1347476

[B4] Bartscherer K Pelte N Ingelfinger D Boutros M Secretion of Wnt ligands requires Evi, aconserved transmembrane protein Cell 2006 125 523 533 10.1016/j.cell.2006.04.009 16678096

[B5] Bard F Casano L Mallabiabarrena A Wallace E Saito K Kitayama H Guizzunti G Hu Y Wendler F Dasgupta R Perrimon N Malhotra V Functional genomics reveals genes involvedin protein secretion and Golgi organization Nature 2006 439 604 607 10.1038/nature04377 16452979

[B6] Huang S Laoukili J Epping MT Koster J Hölzel M Westerman BA Nijkamp W Hata A Asgharzadeh S Seeger RC Versteeg R Beijersbergen RL Bernards R ZNF423 is criticallyrequired for retinoic acid-induced differentiation and is a marker of neuroblastoma outcome Cancer Cell 2009 15 328 340 10.1016/j.ccr.2009.02.023 19345331 PMC2693316

[B7] Boutros M Bras LP Huber W Analysis of cell-based RNAi screens Genome Biology 2006 7 R66 10.1186/gb-2006-7-7-r66 16869968 PMC1779553

[B8] Birmingham A Selfors LM Forster T Wrobel D Kennedy CJ Shanks E Santoyo-Lopez J Dunican DJ Long A Kelleher D Smith Q Beijersbergen RL Ghazal P Shamu CE Statistical methods for analysis of high-throughput RNA interference screens Nature Methods 2009 6 569 75 10.1038/nmeth.1351 19644458 PMC2789971

[B9] Rieber N Knapp B Eils R Kaderali L RNAither, an automated pipeline for the statisticalanalysis of high-throughput RNAi screens Bioinformatics 2009 25 678 9 10.1093/bioinformatics/btp014 19168909

[B10] Chung N Zhang XD Kreamer A Locco L Kuan PF Bartz S Linsley PS Ferrer M Strulovici B Median absolute deviation to improve hit selection for genome-scale RNAi screens Journal of Biomolecular Screening 2008 13 149 58 10.1177/1087057107312035 18216396

[B11] Zhang JH Chung TD Oldenburg KR A Simple Statistical Parameter for Use in Evaluation and Validation of High Throughput Screening Assays Journal of Biomolecular Screening 1999 4 67 73 10.1177/108705719900400206 10838414

[B12] Gentleman RC Carey VJ Bates DM Bolstad B Dettling M Dudoit S Ellis B Gautier L Ge Y Gentry J Hornik K Hothorn T Huber W Iacus S Irizarry R Leisch F Li C Maechler M Rossini AJ Sawitzki G Smith C Smyth G Tierney L Yang JY Zhang J Bioconductor: opensoftware development for computational biology and bioinformatics Genome Biology 2004 5 R80 10.1186/gb-2004-5-10-r80 15461798 PMC545600

[B13] Malo N Hanley JA Cerquozzi S Pelletier J Nadon R Statistical practice in high-throughput screening data analysis Nature Biotechnology 2006 24 167 75 10.1038/nbt1186 16465162

[B14] Apache Tapestry 5 http://tapestry.apache.org

[B15] Apache Tomcat 5.5 http://tomcat.apache.org

[B16] Jetty Java webserver http://www.mortbay.org/jetty/

[B17] Virtual Box http://www.virtualbox.org/

[B18] Brideau C Gunter B Pikounis B Liaw A Improved statistical methods for hit selection inhigh-throughput screening Journal of Biomolecular Screening 2003 8 634 647 10.1177/1087057103258285 14711389

[B19] Horn T Arziman Z Berger J Boutros M GenomeRNAi: a database for cell-based RNAiphenotypes Nucleic Acids Research 2007 35 D492 7 10.1093/nar/gkl906 17135194 PMC1747177

